# Genotype–phenotype correlation and natural history analyses in a Chinese cohort with pelizaeus–merzbacher disease

**DOI:** 10.1186/s13023-022-02267-z

**Published:** 2022-03-28

**Authors:** Ruoyu Duan, Haoran Ji, Huifang Yan, Junyu Wang, Yu Zhang, Qian Zhang, Dongxiao Li, Binbin Cao, Qiang Gu, Ye Wu, Yuwu Jiang, Ming Li, Jingmin Wang

**Affiliations:** 1 Department of Pediatrics, Peking University First Hospital, Beijing, 100034 China; 2 Department of Children’s Development and Rehabilitation, Peking University First Hospital, Beijing, 100034 China

**Keywords:** Pelizaeus–merzbacher disease, Genotype, Phenotype, Natural history, Chinese cohort

## Abstract

**Background:**

The natural history and genotype–phenotype correlation of Pelizaeus–Merzbacher disease (PMD) of Chinese patients has been rarely reported.

**Method:**

Patients who met the criteria for PMD were enrolled in our study. Genomic analysis was conducted by multiplex ligation probe amplification (MLPA) and Sanger or whole-exome sequencing (WES). Natural history differences and genotype–phenotype correlations were analyzed.

**Result:**

A total of 111 patients were enrolled in our follow-up study. The median follow-up interval was 53 m (1185). Among PMD patients, developmental delay was the most common sign, and nystagmus and hypotonia were the most common initial symptoms observed. A total of 78.4% of the patients were able to control their head, and 72.1% could speak words. However, few of the patients could stand (9.0%) or walk (4.5%) by themselves. Nystagmus improved in more than half of the patients, and hypotonia sometimes deteriorated to movement disorders. More *PLP1* point mutations patients were categorized into severe group, while more patients with *PLP1* duplications were categorized into mild group (*p* < 0.001). Compared to patients in mild groups, those in the severe group had earlier disease onset and had acquired fewer skills at a later age.

**Conclusion:**

PMD patients have early disease onset with nystagmus and hypotonia followed by decreased nystagmus and movement disorders, such as spasticit. Patients with *PLP1* duplication were more likely to be categorized into the mild group, whereas patients with point mutations were more likely to be categorized into the severe group.

**Supplementary Information:**

The online version contains supplementary material available at 10.1186/s13023-022-02267-z.

## Background

Pelizaeus–merzbacher disease (PMD) is an X-linked recessive hypomyelination disorder. The prevalence of PMD is approximately 1.45 in 100,000 male live births in Japan [1] and 0.13 in 100,000 live births in Germany [2]. As the most common form of hypomyelinating leukodystrophy, PMD is characterized by a broad range of neurological disorders, including nystagmus, hypotonia, moderate-to-severe developmental delay, with or without seizures, and ataxia [3–5]. Boespflug-Tanguy and colleagues proposed a five-grade classification [5–7] of PMD clinical phenotypes based on the patient's best motor achievement (form 0 to form 4); the most severe disease corresponds to form 0 (connatal PMD), and mild cases (classic PMD) are classified as forms 2 and 3. The symptoms of connatal patients include low motor or cognitive skills and disease onset at an early age; most of these patients also have dystonia, seizures and other neurological symptoms. The patients in the classic group acquire more skills but have symptoms of hypotonia. Although nystagmus decreases with disease progression, other motor and cognitive disorders develop [8].

Genomic mutation analysis has revealed that mutations in the proteolipid protein 1 gene (*PLP1*, NM_001128834.20), which are located on Xq22.2, cause PMD [9, 10]. The most common mutation in PMD patients is *PLP1* duplication (60–70%); *PLP1* point mutations are less common (15–20%). Overall, different mutations correspond to different phenotypes. With *PLP1* duplications often causing form 1 and form 2, the phenotype of *PLP1* point mutations ranges across the entire spectrum from the most severe (form 0) to the mildest (form 4) form [2, 5]. Individuals who carry deletion and nonsense/frameshift mutations that constitute a null mutation always display a mild severe phenotype of form 3 and form 4 [11]. Due to the lack of accessibility to genomic therapy, several treatments, such as cholesterol supplementation, ketogenic diet, and iron chelators, have been identified as viable alternatives in clinical application [12–14]. Regardless, more studies should be conducted to demonstrate whether pharmacologic therapy can be further applied.

As the phenotype and genotype have become more clear in recent years, an increasing number of PMD cases have been recorded in our database. Various PMD studies have been performed, among which two PMD follow-up studies have demonstrated the genotype and phenotype of PMD [15, 16]. Nevertheless, no large cohort study has described the natural history of PMD and genotype–phenotype correlation among Chinese PMD patients. This study included a large cohort of Chinese PMD patients and analyzed genotype–phenotype correlation and natural history in a Chinese cohort with pelizaeus–merzbacher disease, providing a foundation for the diagnosis and treatment of PMD.

## Results

### General information of the patients and follow-up

**Fig. 1:**
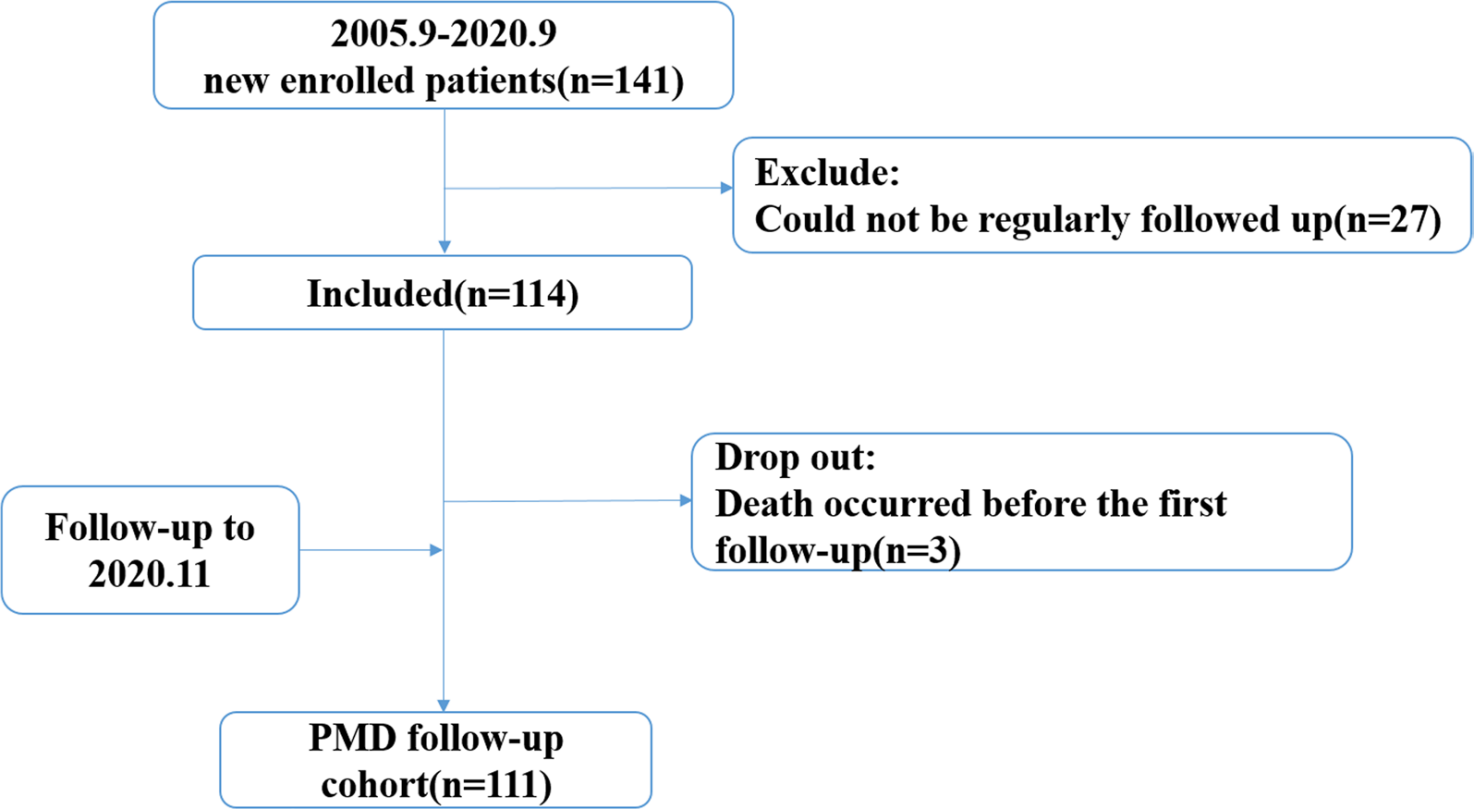
Follow-up study design

A total of 141 patients were genetically diagnosed with PMD, and 111 were followed up (105 males and 6 females) from 2005 to 2020 at Peking University First Hospital. The median follow-up interval was 53 m (1185); 30 patients were excluded based on the exclusion criteria (Fig. 1). The parents were all nonconsanguineous. However, for 12.6% (14/111) of the cases, more than one affected family member was regarded as having developmental delay (DD). Pt99 and Pt100, Pt105 and Pt106 were brothers, as were Pt111 and Pt112. 6 patients (5.4% 6/111) had died by the time of the last follow-up.

Three follow-up studies were conducted in March 2012, March 2017, and November 2020: 45 patients for the first and 31 for the second, with a 60-month interval between the first and second follow-ups; 75 patients for the third follow-up, with a 44-month interval between the second and third follow-ups. 7 patients were seen four times, with a mean follow-up interval of 139.8 ± 32.6 m; 25 patients were seen three times, with a mean follow-up interval of 97.8 ± 31.5 m; 79 patients were seen two times, with a mean follow-up interval of 49.2 ± 29.5 m (Additional file 1: Table S2). Some patients were lost to follow-up due to failure to make contact.

### Genotype features of Chinese PMD

All 141 patients carried a *PLP1* mutation, namely, 110 duplications (Pt1-Pt110) and 31 (Pt111–Pt141) point mutations. The patients showed hemizygosity or heterozygosity, with 12 de novo mutations (5 duplications, 7 point mutations). Twenty-six mutations have been reported, and 3 patients (Pt116, Pt117, Pt118) harbored the same F32L mutation as previously reported in another PMD patient [5]. The P216S mutation (Pt134) was found in two other studies [5, 17]. Twenty-nine of 31 point mutations were missense mutations, 2 were deletions (E38_L40del, L224del), and 2 mutations (C33R, L239V) were missense amino acid changes occurring at the same position as another pathogenic missense change. Mutations were classified as pathogenic, likely pathogenic or VUS via in silico programs (Table 1).

**Table 1:** The information and molecular finding of the point mutation in our cohort

Case	Nucleotide change	Amino-acid change	Parental derivation	Novel/reporter (frequency)	PCS	Location	Pathogenicity prediction				ACMG
							Polyphen2	SIFT	gnomAD	CADD	
Pt111 Pt112	c.62C > T	p.(A21V)	Maternal	N	NA	A	PD	D	–	26.8	LP (PM2 + PP1 + PP2 + PP3 + PP4)
Pt113	c.73T > C	p.(C25R)	Maternal	N	NA	A	PSD	D	–	25.6	VUS (PM2 + PP2 + PP3 + PP4)
Pt114	c.82G > C	p.(G28R)	Maternal	R	2	A	PD	D	–	26.6	LP(PS1 + PM2 + PP2 + PP3 + PP4)
Pt115	c.92T > C	p.(L31P)	Maternal	R	1	A	PD	D	–	26.4	LP(PS1 + PM2 + PP2 + PP3 + PP4)
Pt116	c.94T > C	p.(F32L)	Maternal	R	3	A	PD	D	–	26.4	LP(PS1 + PM2 + PP2 + PP3 + PP4)
Pt117	c.94T > C	p.(F32L)	De novo	R	1	A	PD	D	–		P(PS1 + PS2 + PM2 + PP2 + PP3 + PP4)
Pt118	c.96C > G	p.(F32L)	De novo	R	0	A	PD	D	–	24.4	P(PS1 + PS2 + PM2 + PP2 + PP3 + PP4)
Pt119	c.97T > C	p.(C33R)	Maternal	R	0	A	PD	D	–	26.2	LP(PS1 + PM2 + PP2 + PP3 + PP4)
Pt120	c.111_119 delTGAAGCCCT	p.(E38_L40del)	Maternal	N	1	A–B loop	/	/	–	–	VUS(PM2 + PP4)
Pt121	c.353C > G	p.(T118R)	Maternal	R	3	PLP-S	PD	T	-	23.6	LP(PS1 + PM2 + PP2 + PP3 + PP4)
Pt122	c.467C > T	p.(T156I)	De novo	R	2	C	PD	D	–	25.1	P(PS1 + PS2 + PM2 + PP2 + PP3 + PP4)
Pt123	c.467C > T	p.(T156I)	Maternal	R	NA	C	PD	D	–		LP(PS1 + PM2 + PP2 + PP3 + PP4)
Pt124	c.469T > C	p.(Y157H)	Maternal	R	2	C	PD	D	–	27.7	LP(PS1 + PM2 + PP2 + PP3 + PP4)
Pt125	c.508T > C	p.(S170P)	Maternal	R	1	C	PSD	D	–	26.0	LP(PS1 + PM2 + PP2 + PP3 + PP4)
Pt126	c.515T > C	p.(V172A)	De novo	R	1	C	B	D	–	23.6	P(PS1 + PS2 + PM2 + PP2 + PP3 + PP4)
Pt127	c.517C > T	p.(P173S)	Maternal	R	1	C	PD	D	–	26.5	LP(PS1 + PM2 + PP2 + PP3 + PP4)
Pt128	c.535A > C	p.(N179H)	Maternal	R	NA	C–D loop	PD	D	–	24.4	P(PS1 + PM1 + PM2 + PP2 + PP3 + PP4)
Pt129	c.552C > G	p.(C184W)	Maternal	R	NA	C–D loop	PD	D	–	25.4	P(PS1 + PM1 + PM2 + PP2 + PP3 + PP4)
Pt130	c.613A > G	p.(R205G)	Maternal	R	2	C–D loop	PD	D	–	26.2	P(PS1 + PM1 + PM2 + PP2 + PP3 + PP4)
Pt131	C.614G > A	p.(R205K)	Maternal	R	4	C–D loop	PD	D	–	27.8	P(PS1 + PM1 + PM2 + PP2 + PP3 + PP4)
Pt132	c.623G > A	p.(G208D)	Maternal	R	2	C–D loop	PD	D	–	33	P(PS1 + PM1 + PM2 + PP2 + PP3 + PP4)
Pt133	c.623G > T	p.(G208V)	Maternal	R	NA	C–D loop	PD	D	–	34	P(PS1 + PM1 + PM2 + PP2 + PP3 + PP4)
Pt134	c.646C > T	p.(P216S)	De novo	R	5	C–D loop	PD	D	–	26.5	P(PS1 + PS2 + PM2 + PP2 + PP3 + PP4)
Pt135	c.646C > A	p.(P216T)	Maternal	N	1	C–D loop	PD	D	–	25.6	LP(PS1 + PM2 + PP2 + PP3 + PP4)
Pt136	c.670_672 delCTT	p.(L224del)	Maternal	N	NA	C–D loop	/	/	–	–	VUS(PM2 + PP4)
Pt137	c.709T > G	p.(F237V)	De novo	R	0	D	B	D	–	24.6	P(PS1 + PS2 + PM2 + PP2 + PP3 + PP4)
Pt138	c.715C > G	p.(L239V)	Maternal	R	1	D	PD	D	–	27.2	LP(PS1 + PM2 + PP2 + PP3 + PP4)
Pt139 Pt140	c.718T > C	p.(F240L)	Maternal	R	1	D	PD	D	–	28.5	LP(PS1 + PM2 + PP2 + PP3 + PP4)
Pt141	c.743C > A	p.(A248E)	De novo	R	1	D	PD	D	–	27.3	LP(PS1 + PM2 + PP2 + PP3 + PP4)

*R* reported, *N* novel, *PCS* phenotype classification score, *NA* not available due to lose to follow up, *PD* probably_damaging, *PSD* possibly_damaging, *B* benign, *D* deleterious, *T* tolerated, not found in the database

### Natural history

#### Disease onset

**Fig. 2:**
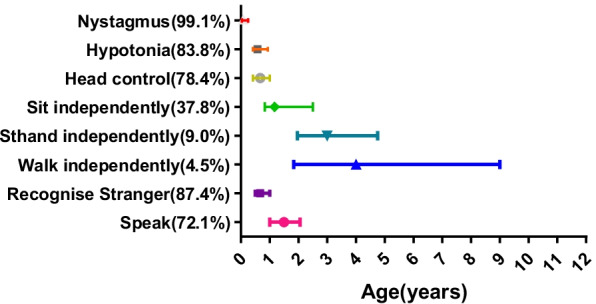
Development of clinical features over time in PMD patients. The x axis indicates the age of onset (years) and the y axis indicates the clinical features. Percentages denote the proportion of patients with a given clinical feature. Individual patient ages are displayed with the median. The lower and upper hinges correspond to the 25th and 75th percentiles

The development of clinical features over time is demonstrated in Fig. 2. The median age of disease onset was 1 m (0, 15). Development delay was the chief sign in all of the children (100%), with nystagmus (99.1% 110/111) and hypotonia (83.8% 93/111) being the most common initial symptoms observed. The median age of nystagmus onset was 1 m (0, 24), and the median age of hypotonia onset was 7 m (2, 26). Symptoms of stridor (31.5% 35/111), swallowing disorder (17.1% 19/111), and respiration difficulty (3.6% 4/111) were less common.

#### Disease progression

Developmental milestones were all delayed, with 78.4% (87/111) of the patients being able to control their head, 37.8% (42/111) sitting independently, 9.0% (10/111) standing by themselves, and 4.5% (5/111) walking independently. The median ages for head control, sitting, standing and walking independently were 8 m (3, 71), 14.5 m (6, 96), 36 m (13, 84), and 48 m (15, 120), respectively. A total of 87.4% (97/111) of the patients recognized strangers at a median age of 8 m (3, 60), and 72.1% (80/111) of the patients spoke words at a median age of 18 m (6, 86). During the three follow-ups, nystagmus was decreased in more than half of the patients (58.2% 64/110) at the median age of 24 (4, 260), disappearing in 10.9% (12/110) of them. Other movement disorders were found, including joint contracture (24.7%, 23/93), spasticity (5.4%, 5/93). Seizures were present in 7.2% (8/111) of the patients until the last follow-up, and one patient died because of epileptic seizures at the age of 7 years.

#### Clinical characteristics in different subgroups

Based on motor and cognitive development and neurological symptoms, 25.2% (28/111) of the patients were categorized into group A, with 50.5% (56/111) in the group B and 24.3% (27/111) in the group C. The clinical information of all 111 patients is described in Additional file 1: Table S3. Comparing the natural history and disease progression of the three subgroups (Table 2), the onset of nystagmus occurred earlier in group A than group B (*p* = 0.016). More patients in group A than in the groups B and C displayed hypotonia at disease onset (*p* = 0.005). For disease progression, fewer patients in group A acquired the ability to speak, spoke later and showed progression to stridor (*p* = 0.011) and brainstem dysfunction than in group B (*p* < 0.001) and C (*p* < 0.001). Moreover, more patients in group C acquired head control at an earlier age and were able to sit by themselves than patients in group B and C (*p* < 0.001). In addition, patients in group C acquired sitting ability earlier than in group B (*p* = 0.027).

**Table 2:** The clinical phenotype comparison between different clinical subgroup

	Connatal group (n = 28)	Transitional group (n = 56)	Classic group (n = 27)	*p* value
*Disease onset*				
Nystagmus, n (%)	28 (100)	56(100)	26(96.3)	0.208
Onset age (range)	0.85 m (0, 12)	1 (0, 24)	2 m (0, 16)^b^	0.016*
Hypotonia, n (%)	18 (64.3)^a^	50 (89.3)	25 (92.6)^b^	0.005*
Onset age (range)	6.5 m (2. 18)	8 m (3, 26)	7 m (2, 18)	0.381
*Disease progression*				
Nystagmus decrease	13 (46.4)	32 (57.1)	19 (73.1)	0.136
Decrease age (range)	12 m (4, 96)	26 m (8, 79)	24 m (7, 260)	0.291
Stridor	15 (53.6)^a^	15 (26.8)	5 (18.5)^b^	0.011*
*Motor milestone*				
Head control, n (%)	12 (42.9)	48 (85.7)	27 (100)	< 0.001*
Acquire age (range)	11 m (4, 71)	10 (3, 60)^c^	5 (3, 18)^b^	< 0.001*
Sit, n (%)	4 (14.3)	14 (25)^c^	26 (88.9)^b^	< 0.001*
Acquire age (range)	47 (7, 96)	33 (8, 86)^c^	12 (6, 84)	0.027*
Stand, n (%)	1 (3.6)	4 (7.1)	5 (18.5)	0.121
Acquire age (range)	13 (13, 13)	41 (26, 48)	36 (16, 84)	0.273
Walk, n (%)	1 (3.6)	2 (3.6)	2 (7.4)	0.705
Acquire age (range)	15 (15, 15)	38.5 (29, 48)	108 (96, 120)	0.165
*Cognitive milestone*				
Recognize stranger, n (%)	21 (75.0)	51 (91.1)	25 (92.6)	0.072
Acquire age (range)	8 (3, 48)	8 (4, 60)	8 (4, 36)	0.955
Speak, n (%)	11 (39.3)^a^	44 (78.6)	25 (92.6)^b^	< 0.001*
Acquire age(range)	36 (12, 72)^a^	19 (6, 86)	12 (6, 30)^b^	0.002*
*Other neurological finding*				
Joint contracture, n (%)	8 (28.6)	10 (17.9)	5 (18.5)	0.494
Spasticity tetraparesis, n (%)	0	4 (7.1)	1 (3.7)	0.322
Pyramidal signs, n (%)	3 (10.7)	3 (5.4)	6 (22.2)	0.068
Brainstem dysfunction, n (%) (Swallowing difficulty, respiration dysfunction)	15 (53.6)^a^	8 (14.3)	0 (0)^b^	< 0.001*
Ataxia, n (%)	2 (7.1)	3 (5.4)	2 (7.4)	0.917
Seizure, n (%)	4 (14.3)	4 (7.1)	0	0.123

*Significant difference between three groups

^a^Significant between connatal and transitional group was significant

^b^Significant between connatal and classis group was significant, ^c^Significant between transitional and classic group was significant

#### Motor and cognitive skill evaluation

At the first follow-up, no patients’ motor scale was classified as GMFCS I; GMFCS II was indicated in 1 (2.6%, n = 39), GMFCS III in 6 (15.4%), GMFCS IV in 10 (25.6%), and GMFCS V in 22 (56.4%). At the third follow-up, after 8.7 years, motor skills were re-evaluated in 75 patients and classified as GMFCS I in 2 (2.6%), GMFCS II in 9 (12.0%), GMFCS III in 12 (16.0%), GMFCS IV in 17 (22.7%), and GMFCS V in 35 (46.7%). Furthermore, among the 39 patients who followed-up two times with the motor scale GMFCS, 51.3% (20/39) patients classified as GMFCS IV or V, 20.5%(8/39) patients deteriorated to a severer motor disability displayed with a higher GMFCS level. 28.2% (11/39) patients achieved more motor skills thus acquired a lower GMFCS level.

At the third follow-up, none of patients in group A were classified with GMFCS I, II, or III. In contrast, GMFCS V was found in 83.3% of the patients in group A and 33.3% of the patients in group C. Higher GMFCS levels were more likely to be observed in group A, with lower levels more likely in group C (*p* < 0.001, G = -0.550).

There was a correlation between clinical classification and IJMSSSLAS raw scores; specifically, a patient with a milder phenotype had a higher raw score (Kendall's tau_b = 0.280 *p* = 0.001). However, there was no correlation between clinical classification and S–M scale level (*p* = 0.058).

#### Brain MRI

All 111 patients underwent brain MRI at their first visit or follow-up visit. Hypomyelinating leukodystrophy with hyperintensity in brain white matter on T2WI MRI scans was the dominant characteristic.

**Fig. 3:**
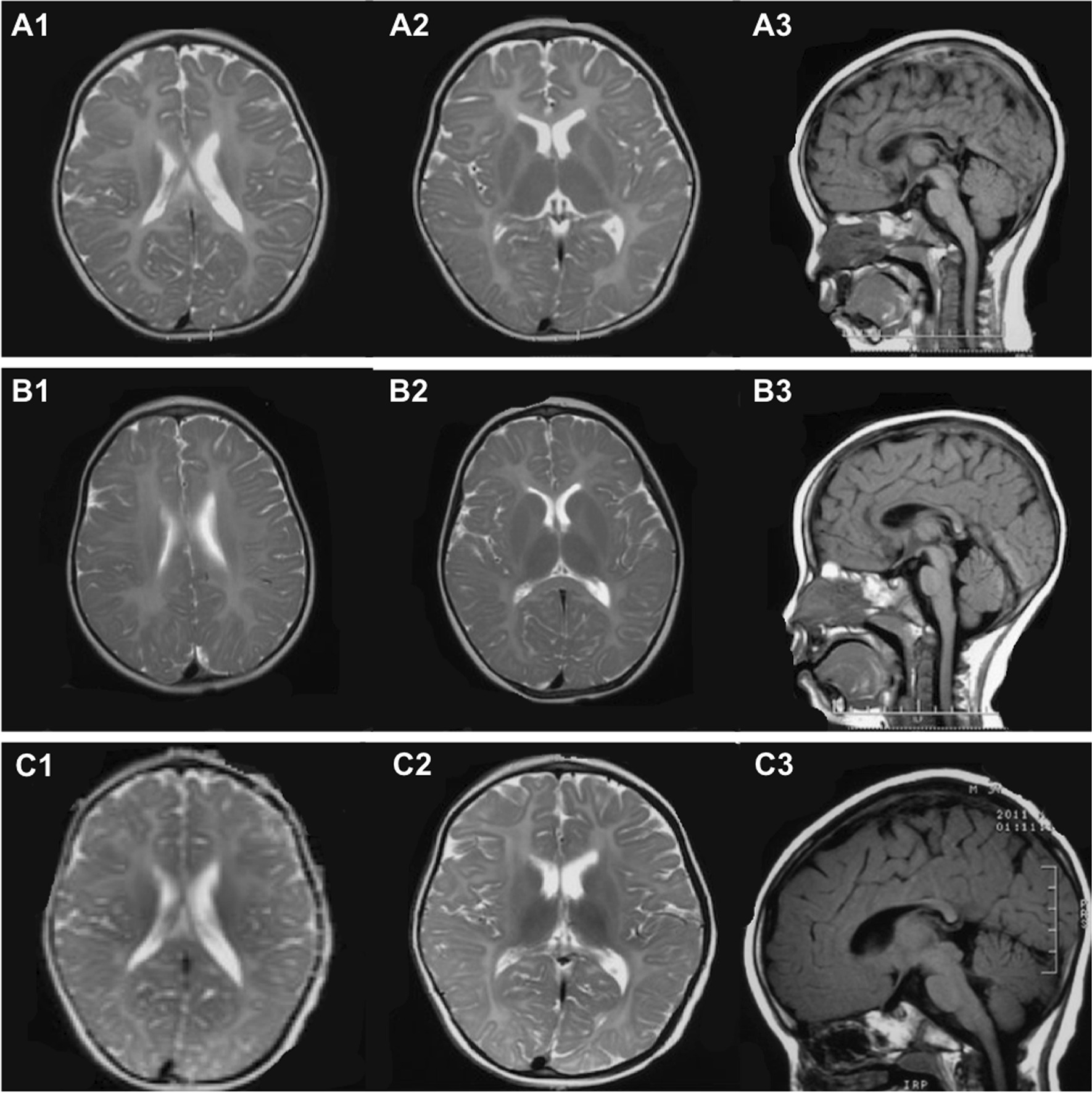
Brain MRI of patients with PMD. **A1**–**A3**, **B1**–**B3**, and **C1**–**C3** represent the comparison of Pt28 brain images at 14 m, 30 m and 47 m, respectively. **A1**–**A2**, **B1**–**B2**, **C1**–**C2** Diffuse hyperintensity in white matter presented in axial T2WI. **A3**, **B3**, **C3** show atrophy of the corpus callosum on sagittal T1WI

MRIs were obtained at the follow-up for 31 patients, and 100% (31/31) of them showed corpus callosum atrophy (Fig. 3); 29.0% (9/31) displayed supratentorial brain atrophy and 3.2% (1/31) cerebellum atrophy. The mean age of corpus callosum atrophy was 33.1 ± 21.8 m in all 31 patients, and 9 patients exhibited supratentorial brain atrophy at a mean age of 26.4 ± 16.0 m; only 1 patient exhibited cerebellum atrophy, at 91 m.

### Genotype–phenotype correlation

Among the 23 patients carrying *PLP1* point mutation in the follow-up study, 60.9% (14/23) were in group A, 30.4% (7/23) in group B and 8.7% (2/23) in group C (Table 1). The percentages of patients in groups A, B, C with *PLP1* duplication were 15.9% (14/88), 55.7% (49/88) and 28.4% (25/88), respectively. Patients with *PLP1* point mutations were more likely to be classified into group A (*p* < 0.001); patients with *PLP1* duplications were more likely to be classified into group B (*p* < 0.001).

Twelve de novo* PLP1* mutations were found in our cohort, with five duplications and seven point mutations (Table 1). Mutations of A21V (Pt111, Pt112) and F32L (Pt116, Pt117, Pt118), T156I (Pt122, Pt123), F240L (Pt139, Pt140) were found to be recurrent.

Three patients in five de novo* PLP1* duplication mutations are females. Two of them (Pt79 and Pt108) presented with severe phenotype which classified into group A, the third female (Pt65) died at 18 months due to respiratory failure; The other two males were classified into group B (Pt96) and group C (Pt20). Patients with F32L was classified into different clinical subgroups with the same mutation (Table 1). Pt116 was a female who carried an F32L mutation inherited from her mother and exhibited a mild phenotype which classified into Group B. Her mother was identified as a case of mosaicism in the follow-up study. Pt117 and Pt118 were males with de novo F32L, and their clinical phonotype were classified as group A; Pt118 died at 7 years old. Six mutations (R205G, R205K, G208D, G208V and P216S, P216T) were observed in three positions of PLP1*,* and these 6 patients displayed different phenotypes (Table 1). The patients who harbored F240L displayed a severe phenotype in group A. There also was one female with a de novo* PLP1* deletion (Pt4), she acquired head control at 17 months, and concomitant symptoms of nystagmus and hypotonia were also diagnosed.

**Fig. 4:**
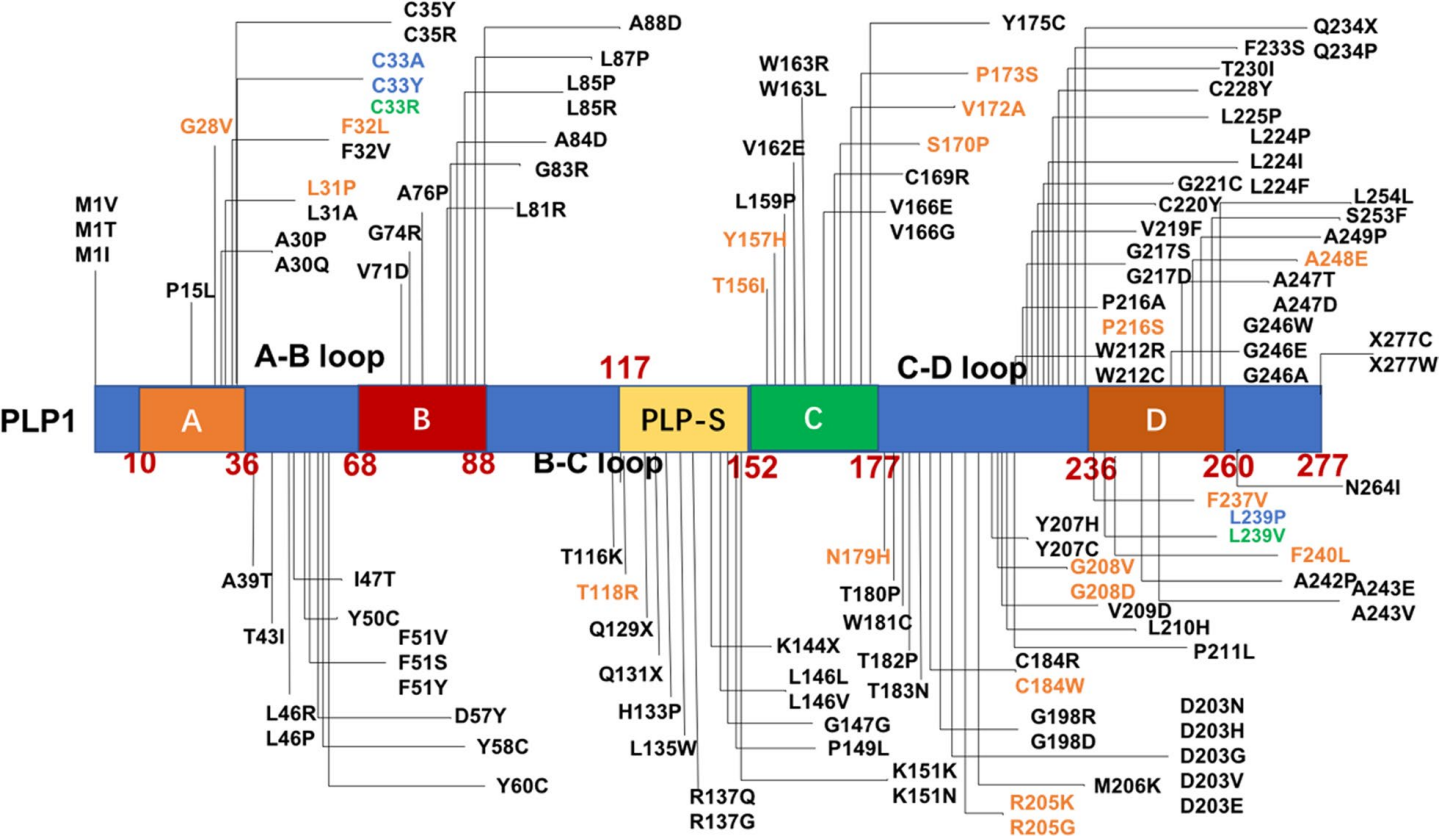
PLP1 structure and all mutations reported in the literature. A, B, C, and D indicate the four transmembrane domains and three loops between them (A–B loop, B–C loop, C–D loop). The PLP-special section (PLP-S) is located near the C domain in the B–C loop. Black mutations had been reported in literatures; yellow mutations were detected in our patients and had been reported in literatures. Green mutations were detected in our patients and hadn’t been reported in literatures. Blue mutations are located at the same position as another pathogenic missense change

PLP1 is a transmembrane protein with 4 transmembrane (A, B, C, D) domains interspersed with 3 connection loops (A–B loop, B–C loop, C–D loop). A special domain of the PLP1 section (PLP-S) is located in the B–C loop (Fig. 4). 12, 10, 13 and 15 mutations have been reported in the A, B, C, and D domains, and 12, 15 and 42 mutations in the A–B, B–C, and C–D loops, respectively. Nine of the mutations in our cohort are located in the A domain, one in the A–B loop, one in the PLP-S domain, six in the C domain, five in the D domain, and nine in the C–D loop.

## Discussion

PMD is one of the most prevalent X-linked white matter disorders. A *PLP1* duplication or point mutation can lead to this disorder [18]. Previous studies have shown that 60–70% of mutations comprise *PLP1* duplications; 15–20% alterations are point mutations involved in *PLP1*-related disorders, insertions or deletions [16]. Large deletions of *PLP1* have been reported less frequently. Our PMD cohort had higher rates of *PLP1* duplications (74.7%) and point mutations (22.0%), perhaps because our study did not include *PLP1* null mutations or splice site mutations that most often lead to an SPG2 disorder [5, 19]. Affinity analyses have shown that de novo mutations in *PLP1* duplications are much rarer than are point mutations [5]. De novo* PLP1* duplications were found in 4.5% (5/110) of the patients in our cohort, whereas 22.6% (7/31) of the patients carried de novo* PLP1* missense mutations. The proportion of *PLP1* duplications and point mutations occurring de novo in our cohort was lower than that in the Mimault et al. study [20]. This is partly because previous studies also included splice site mutations. Additionally, the emergence of MLPA has provided a method for detecting small-segment duplications and deletions that is highly convenient. Indeed, more maternal carriers can be easily identified with MLPA.

PMD caused by *PLP1* mutations is characterized by developmental delay, nystagmus, hypotonia and other neurological symptoms. PMD onset occurs early, with the symptom of nystagmus or hypotonia between the 1st and 8th months of life [16], in accordance with our result of 1 m (1, 15). However, the patients’ first visit time was 12 m (1336), which was much later than disease onset. Mostly due to although the majority of PMD patients showed retardation of psychomotor and neurological symptoms, they still acquired a number of abilities in their first years, with most at one or more years of age developed into intelligent disability or developmental delay [5, 15]. Most patients acquired the ability of head control (78.4%) and speech (72.1%); fewer patients could sit (37.8%), and fewer could stand (9.0%) or walk (4.5%) by themselves. This is in accordance with the fact that most patients with PMD cannot stand independently but may be able to speak with form 2 and form 3 [5]. As the disease progresses, nystagmus decreases or even stops, which was also reported by Torii et al. [8]. Developmental delays occurred in almost all of the patients during their lifetime.

Different genotypes have been described for PMD, and the phenotype of PMD also varies in different clinical subgroups. In contrast to Boespflug-Tanguy and colleagues’ study, which classified PMD patients only by their best motor skills, our study classified PMD patients into three groups based on disease onset age, motor skills, cognitive skills and other neurological symptoms in disease progression [21]. In the studies of Cailloux and Shimojima, 42% and 30% of patients with *PLP1* duplications had form 2 and form 3 of the disease, respectively; 72% of the patients had mild PMD, and none displayed form 4 or form 0 duplications [5, 22]. In our cohort, the ratio of patients in group A: group B: group C was 1:2:1 (25.2%:50.5%:24.3%). Overall, our study included more severe patients. Because the most severe form of PMD is exclusively caused by point mutations, conservation of mutation correlates strongly with the severe phenotype [8]. The pathogenic point mutations in our study are discussed below. Comparison of natural history between different subgroups revealed an earlier onset and slower improvement in the group A than in group B or group C. Motor and cognitive development were evaluated by scales, and the results also showed higher motor skill level and IJMSSSLAS raw scores group B and groups C than in group A, but there was no correlation between clinical classification and S–M scale level (*p* = 0.058). Because PMD patients’ skills develop slower than those in normal children, there was no significant difference in IJMSSSLAS level among the PMD subtypes when taking age into consideration. These phenotypes were partly described in the Torii et al. study [8]. Our study’s PCS system combines Cailloux’s and Hurst’s methods in an improved system which even takes patients’ neurological phenotypes into consideration (also added in the Additional file 1: Table S2). We believe this allows for better evaluation of PMD patients’ phenotypes. For the first time, our study demonstrates the motor and cognitive skills of three subtypes of PMD by scales and further lays a foundation for the precise diagnosis and treatment of the disease.

The phenotype of PMD also has relationships with genotype. Patients harboring *PLP1* point mutations displayed higher connatal type levels, and more mild patients in group B were found with *PLP1* duplication. The type of point mutation is considered related to the severity of PMD [5]. For example, twice as much PLP1 was detected in patients carrying *PLP1* duplication as in controls [22–24]. Indeed, dosages and breakpoints or structures of gained segments correlate strongly with the clinical severity of PMD [21]. A demyelination disorder mouse model named “jimpy” is similar to human PMD [25], With a lack of 208–232 amino acid regions in jimpy mouse, PLP1 dysfunction and oligodendrocyte cell death were observed [26]. This region is located in the C–D loop, and more mutations in this region (39%) were reported in the Cailloux et al. study. 29.0% (9/31) of mutations in our study were detected in the C–D loop, a domain with the most mutations in our study. Mutations in this section can cause both severe and mild PMD in both previously [5] and our study (Table 1). Two missense mutations (C33Y and S170P) are in hydrophobic transmembrane spans and may interfere with correct folding of the polypeptide. C33Y has not been shown to be associated with severe PMD, and S170P correlates with a mild clinical course [27], in accordance with our results. The mutation of T118R in PLP-S is responsible for a mild phenotype, which in accordance with nearby T115K has been classified as a form of SPG [28]. Another mutation, T156I, found in our study is located in a transmembrane domain, but this mutation appears to cause little disruption [29, 30]; therefore, the patients (Pt122, Pt123) with this mutation were categorized into group B. A proline mutation results an α-helix turning and thus alteration of the PLP1 structure [31], which can explain why P173S corresponds to group A. Moreover, different PLP1 mutants exhibited distinct localizations, and the pathogenic mechanisms of F32L, T118R, T156I, P173S, R205K, G208V and G208D were demonstrated in our other study [32].

6 females were diagnosed as PMD in our study. As we all know, carrier females of *PLP1* duplication are usually asymptomatic. However, X-chromosome inactivation (XCI) studies showed a subtle inactivation were found in one female patient’s (Pt65) X-chromosome, whereas her mother had a higher level of X-inactivation in our study. Pt116’s mother was proved to be a germline mosaic who transmitted the mutation to her daughter. The mosaicism in the heterozygous females also appears due to the effect of XCI. Once the X-chromosome is inactivated it remains so through subsequent cell divisions and differentiation. The mother has more wild type cell lines so she displayed with normal phenotype. After all, XCI plays an important role in female PMD patients’ phenotype. Females with a de novo* PLP1* deletion (Pt114) displayed a mild phenotype, previous studies have also indicated that *PLP1* deletion is mostly related to a mild phenotype of PMD [33, 34].

This study has several limitations. First, for adaptive behavior evaluation, the IJMSSSLAS was not designed for children with such limited function. Many questions were simply not pertinent to the sample studied, and there was certainly a floor effect when looking at physical functioning because these questions addressed activities that most PMD patients will never be able to attain. Second, because of the patients’ difficulties with respect to motor skills, face-to-face follow-up was impossible; therefore, some symptoms, such as spasticity and pyramidal signs, could not be measured with optimal accuracy, the detail of other neurological findings were attached in Additional file 1: Table S2. In addition, despite few patients with *PLP1* splice site mutations who presented with SPG2 in our outpatient department, there were no follow-up procedures because they were enrolled after our third follow-up.

## Conclusion

Our study examined a Chinese PMD cohort and revealed that these patients have a natural history of disease onset with nystagmus and hypotonia. Most patients can acquire the skills of head control and speech; fewer patients can sit on their own, and even fewer can stand and walk on their own. Patients with *PLP1* point mutations are likely to display a connatal phenotype, whereas patients with *PLP1* duplications are likely to display a transitional phenotype. Compared to transitional and connatal PMD patients, more classic PMD patients acquire head control, sitting and speaking abilities at an earlier age, without developing stridor and brainstem dysfunction. With regard to lifespan, the oldest patient was 40 years old; most patients can live to the first decade. Overall, our study provides a foundation for further precise diagnosis and pharmacologic therapy for PMD patients.

## Methods

### Patients

This was a prospective cohort study with retrospective data analysis. A total of 141 patients diagnosed with PMD were enrolled in the outpatient department of Peking University First Hospital from September 2005 to September 2020. The patients met the following criteria: (1) presenting the critical symptoms of PMD, i.e., early onset nystagmus, hypotonia, and hypomyelination on brain MRI; (2) genetic test results confirming *PLP1* mutation; (3) informed consent forms signed. The exclusion criteria for this study included a diagnosis of leukodystrophies caused by toxic injury, infection or demyelinating disorders [35] or failing to meet the inclusion criteria listed above. All patients were of Han ethnicity.

Assessment was performed by a pediatrician, specifically the neurological pediatrician, at our facility. Every follow-up consultation was recorded and examined by a senior pediatric neurologist. The phenotype classification score (PCS) was evaluated separately by at least two pediatricians and would only be reported if the two assessments were consistent. For controversial PCS results, a third pediatrician was invited to provide a reassessment, after which we compared the three PCSs and recorded the final score.

Our study was approved by the clinical research ethics committee of Peking University First Hospital. Written informed consent was obtained from the families of all the enrolled patients.

### Genotype analysis

Genomic DNA was extracted from peripheral venous blood leukocytes of the children and their parents. The sequence of *PLP1* was obtained from the UCSC genome Bioinformatics database (*PLP1*: NM_001128834.20). Various *PLP1* copy numbers were detected by multiplex ligation probe amplification (MLPA). For patients with negative sequencing results, *PLP1* point variations were identified by Sanger or whole-exome sequencing (WES). A novel variation in *PLP1* was examined by SIFT (http://sift.jcvi.org/), Polyphen-2 (http://genetics.bwh.harvard.edu/pph2/), and gnomAD (http://gnomadold.broadinstitute.org), CADD (https://cadd.gs.washington.edu/download) and categorized according to American College of Medical Genetics (ACMG) guidelines [36].

### Information collection and follow-up study

The patients’ information, including basic information (age, sex), developmental milestones, initial symptoms and symptoms during progression, brain MRI, family history and therapy involvement, was collected at their visit.

### Follow-up study

Three follow-up studies were conducted in March 2012, March 2017 and November 2020. Patients were followed up 1–3 times and interviewed via outpatient visits, telephone or WeChat. Clinical analyses focused on natural history and differences between motor impairment and cognitive evolution.

The patients’ motor skills were evaluated by Gross Motor Function Classification System (GMFCS) and divided into five levels (I–V) [37]. Their adaptive behavior was measured based on standard scores of Infant-Junior Middle School Social Adaptive Capacity Scale (IJMSSSLAS). The scale includes 132 items, and one point is awarded for each achieved item. Raw scores can be transformed into a standardized score. Normal level, borderline level, mildly borderline level, median abnormal level, severely abnormal level and profoundly abnormal level correspond to standardized scores ≥ 10, 9, 8, 7, 6 and 5, respectively [38, 39].

### Statistical analysis

Based on the progression of motor and intelligence disability, the clinical severity of each patient was investigated according to the phenotype classification score (PCS) [5, 22, 40, 41] (Additional file 1: Table S1). PCSs ranged from 0 to 5, the lower the score, the fewer abilities a patient would achieve and displayed with more neurological symptoms; scores of 0–1, 2–3 and 4–5 were classified as group A, group B and group C, which corresponded to a sever, mild and milder PMD phenotype, respectively[21]. The natural history of the clinical phenotype, GMFCS, and IJMSSSLAS scores were calculated for each group. Enumeration data are expressed as frequencies and percentages. Measurement data are expressed as the median (range) for unmorally distributed variables or the mean with SD for normally distributed variables. The chi-square test and Fisher’s exact test were used to compare enumeration data. The Kruskal–Wallis test was employed to compare continuous variables among the connatal, transitional, and classic groups. Kendall’s tau-b correlation was utilized to assess the relationship between IJMSSSLAS results and PMD subtypes. Significant differences were indicated when *p* < 0.05. The statistical analysis was conducted with SPSS 20.0 software for Windows.

## Supplementary Information

**Additional file 1. Table S1.** The Phenotype Classification Scores (PCS) used in our study.

## Data Availability

The datasets generated and/or analyzed during the current study are not publicly available but are available from the corresponding author on reasonable request.

## Abbreviations

PMD: Pelizaeus–merzbacher disease
MLPA: Multiplex ligation probe amplification
WES: Whole-exome sequencing
*PLP1*: Proteolipid protein 1 gene
DD: Developmental delay
PLP-S: PLP1 section
PCS: Phenotype classification score
ACMG: American College of Medical Genetics
GMFCS: Gross motor function classification system
IJMSSSLAS: Infant-junior middle school social adaptive capacity scale
XCI: X-chromosome inactivation
