# Supplementary material for: Genotype–phenotype correlation and natural history analyses in a Chinese cohort with pelizaeus–merzbacher disease
Source: Orphanet J Rare Dis. 2022 Mar 28;17:137. doi: 10.1186/s13023-022-02267-z (PMC8962489; doi:10.1186/s13023-022-02267-z)
Supplement: Supplementary file 1 — Additional file 1. Table S1. The Phenotype Classification Scores (PCS) used in our study. [file 13023_2022_2267_MOESM1_ESM.pdf]

Table S1. The Phenotype Classification Scores (PCS) used in our study

Clinical Record		Scoring	
		0	1
Disease onset		0-10 days	>10 days
Milestone	Head Control	≥12 months	<12 months
	Sitting	≥36 months	<36 months
	Simple Language Expression	≥36 months	<36 months
Other Symptoms		Yes	No

Other symptoms include convulsion, dyskinesia, dysphagia, joint contracture, stridor, tremor and retrogress.

Table S2. The patients' disease progression information in our follow-up study

Case	First visit age	First Follow-up (2012.3)			Second Follow-up (2017.3)			Third Follow-up (2020.11)		
		Follow-up Interval	Nystagmus	Neurological findings	Follow-up Interval	Nystagmus	Neurological findings	Follow-up Interval	Nystagmus	Neurological findings
Pt2	17m	71m	+	Hypotonia	131m	+	Hypertonia	175m	better	Hypertonia
Pt3	18m	71m	+	Limb Hypertonia	NA	NA	NA	NA	NA	NA
Pt5	10m	75m	+	Hypotonia	NA	NA	NA	NA	NA	NA
Pt6	23m	57m	+	Hyperreflexia, Pyramidal signs	117	+	Pyramidal signs Hypertonia	161m	+	Hypertonia
Pt8	17m	53m	-(1y5m)	Hypotonia	113m	+	Joint Contracture, Spasticity Tetraparesis	NA	NA	
Pt10	12	47m	-(1y)	Hypertonia, Pyramidal Signs	NA	NA	NA	151m		Joint contracture
Pt11	336m	42m	+	Dystonia, Ataxia, Joint Contracture	NA	NA	NA	146m	better	Hypertonia
Pt12	26m	45m	+	Hypotonia	NA	NA	NA	NA	NA	NA
Pt13	11m	45m	+	Hypertonia	105m	+	Joint Contracture	NA	NA	NA
Pt14	30m	41m	+	Joint Contracture	101m	+	Hypotonia	NA	NA	NA
Pt15	31m	41y	-(6y)	Joint Contracture	NA	NA	NA	NA	NA	NA
Pt16	15m	28m	+	Hypotonia	NA	NA	NA	132m	+	Hypotonia
Pt17	28m	31m	better	Hypotonia	NA	NA	NA	NA	NA	NA
Pt18	8m	21m	+	Hypotonia	NA	NA	NA	NA	NA	NA
Pt19	107m	30m	+	Hypotonia	NA	NA	NA	NA	NA	NA
Pt20	48m	24m	-(2y)	Hypotonia	NA	NA	NA	128m	-	Hypertonia
Pt21	39m	22m	-(4y)	Hypotonia	NA	NA	NA	126m	-	Hypertonia
Pt22	16m	21m	-(2y)	Hypotonia	NA	NA	NA	NA	NA	NA

Case	First visit age	First Follow-up (2012.3)			Second Follow-up (2017.3)			Third Follow-up (2020.11)		
		Follow-up Interval	Nystagmus	Neurological findings	Follow-up Interval	Nystagmus	Neurological findings	Follow-up Interval	Nystagmus	Neurological findings
Pt23	5m	21m	+	Hypotonia	81m	+	Hypotonia	NA	NA	NA
Pt24	21m	5m	better	Poor Tendon Reflexes<br>Pyramidal Signs	65m	+	Joint Contracture	NA	NA	NA
Pt25	17m	12m	-(1y5m)	Poor Tendon Reflexes<br>Pyramidal Signs,<br>Joint Contracture	NA	NA	NA	NA	NA	NA
Pt26	24m	12m	-(1y)	Joint Contracture	72m	+	Ataxia	116m	-	Hypotonia
Pt27	12m	12m	UA	UA	1y9m	+	Hypotonia	116m	better	Hypotonia
Pt28	47m	11m	+	Hypotonia	NA	NA	NA	115m	better	Hypotonia
Pt29	71m	7m	UA	UA	11y1m	better	Joint Contracture	NA	NA	NA
Pt30	47m	8m	+	Hypotonia, Hyperreflexia			Pyramidal Signs	NA	NA	NA
Pt31	126m	8m	better	Joint Contracture	68m	+	Hypertonia, Ataxia	112m	+	Hypotonia
Pt32	5m	7m	+	Hypotonia	NA	NA	NA	NA	NA	NA
Pt33	12m	6m	+	Hypotonia	66m	-(4y)	Hypertonia,<br>Joint Contracture	NA	NA	NA
Pt34	1m	5m	better	hypotonia	NA	NA	NA	NA	NA	NA
Pt35	14m	4m	+	Ataxia	64m	better	Pyramidal Signs,<br>Joint Contracture	108m	+	Hypertonia
Pt36	14m	81m	-(5y)	Hypertonia	141m	-	Spasticity Tetraparesis	185m	-	Hypertonia
Pt37	7m	3m	+	Hypotonia, Ataxia	NA	NA	NA	NA	NA	NA
Pt38	3m	1m	+	Hypertonia	NA	NA	NA	NA	NA	NA
Pt39	6m	1m	better	Hypotonia	NA	NA	NA	NA	NA	NA
Pt40	8m	UA	UA	UA	UA	UA	UA	104	+	Hypotonia

Case	First visit age	First Follow-up (2012.3)			Second Follow-up (2017.3)			Third Follow-up (2020.11)		
		Follow-up Interval	Nystagmus	Neurological findings	Follow-up Interval	Nystagmus	Neurological findings	Follow-up Interval	Nystagmus	Neurological findings
Pt42	43m	UA	UA	UA	65m	-	Hypertonia, Ataxia Joint Contracture	NA	NA	NA
Pt43	9m	UA	UA	UA	UA	UA	UA	98m	better	Hyperreflexia
Pt44	29m	UA	UA	UA	53m	+	Hypotonia, Joint Contracture	NA	NA	NA
Pt45	21m	UA	UA	UA	53m	+	Hypotonia	97m	better	Hypertonia
Pt47	11m	3y4m	+	Hypotonia	78m	+	Hypertonia, Ataxia Joint Contracture	122m	+	Hypertonia, Ataxia, Joint Contracture
Pt52	12m	UA	UA	UA	45m	+	Spasticity Tetraparesis	NA	NA	NA
Pt53	7m	UA	UA	UA	45m	+	Joint Contracture Choreoathetosis	89m	better	Hypotonia
Pt55	10m	UA	UA	UA	42m	+	hypertonia	NA	NA	NA
Pt56	6m	UA	UA	UA	35m	better	Spasticity Tetraparesis Choreoathetosis, Hypertonia	79m	+	Hypertonia
Pt58	4y1m	UA	UA	UA	UA	UA	UA	79m	better	Hypotonia
Pt59	12m	UA	UA	UA	UA	UA	UA	85m	better	Hypotonia
Pt61	6m	UA	UA	UA	UA	UA	UA	77m	+	Hypotonia
Pt62	69m	UA	UA	UA	28m	better	Hypertonia, Ataxia, Spasticity Tetraparesis Joint contracture	72m	better	Hypertonia

Case	First visit age	First Follow-up (2012.3)			Second Follow-up (2017.3)			Third Follow-up (2020.11)		
		Follow-up Interval	Nystagmus	Neurological findings	Follow-up Interval	Nystagmus	Neurological findings	Follow-up Interval	Nystagmus	Neurological findings
Pt63	12M	UA	UA	UA	UA	UA	UA	97m	better	Hypotonia
Pt64	2m	UA	UA	UA	UA	UA	UA	74m	+	-
Pt66	11m	UA	UA	UA	UA	UA	UA	66m	better	Hypotonia
Pt71	19m	UA	UA	UA	UA	UA	UA	55m	better	Hyperreflexia
Pt72	57m	UA	UA	UA	UA	UA	UA	53m	better	Hypotonia
Pt73	34m	UA	UA	UA	9	+	Hypotonia	53m	better	Hypotonia
Pt74	9m	UA	UA	UA	UA	UA	UA	52m	better	Hypotonia
Pt76	5m	UA	UA	UA	UA	UA	UA	49m	better	Hypertonia
Pt77	12m	UA	UA	UA	UA	UA	UA	50m	better	Normal
Pt78	5m	UA	UA	UA	UA	UA	UA	48m	+	Hypotonia
Pt79	9m	UA	UA	UA	8m	+	Hypotonia	52m	better	Hyperreflexia
Pt81	11m	UA	UA	UA	UA	UA	UA	44m	+	Hypotonia
Pt82	7m	UA	UA	UA	UA	UA	UA	40m	+	Hypotonia
Pt83	8m	UA	UA	UA	UA	UA	UA	36m	+	Hypertonia
Pt84	19m	UA	UA	UA	UA	UA	UA	78m	better	Hypotonia
Pt86	29m	UA	UA	UA	UA	UA	UA	13m	better	Hypotonia
Pt87	7m	UA	UA	UA	UA	UA	UA	21m	better	Hypotonia
Pt88	16m	UA	UA	UA	UA	UA	UA	21m	+	Hypotonia
Pt89	14m	UA	UA	UA	UA	UA	UA	9m	better	Normal
Pt90	19m	UA	UA	UA	UA	UA	UA	20m	+	Hypotonia
Pt91	28m	UA	UA	UA	UA	UA	UA	20m	better	Hypertonia
Pt92	6m	UA	UA	UA	UA	UA	UA	40m	better	Hypotonia
Pt93	5m	UA	UA	UA	UA	UA	UA	15m	better	Hypotonia

Case	First visit age	First Follow-up (2012.3)			Second Follow-up (2017.3)			Third Follow-up (2020.11)		
		Follow-up Interval	Nystagmus	Neurological findings	Follow-up Interval	Nystagmus	Neurological findings	Follow-up Interval	Nystagmus	Neurological findings
Pt94	5m	UA	UA	UA	UA	UA	UA	95m	better	Hypotonia
Pt95	31m	UA	UA	UA	UA	UA	UA	5m	better	Hypotonia
Pt96	2m	UA	UA	UA	UA	UA	UA	18m	+	Hypotonia
Pt97	7m	UA	UA	UA	UA	UA	UA	58m	better	Hypotonia
Pt98	3m	UA	UA	UA	UA	UA	UA	50m	+	Hypotonia
Pt99	9m	UA	UA	UA	UA	UA	UA	56m	better	Hypotonia
Pt100	9m	UA	UA	UA	UA	UA	UA	56m	better	Hyperreflexia
Pt101	6m	UA	UA	UA	UA	UA	UA	72m	+	Hyperreflexia
Pt102	1m	UA	UA	UA	UA	UA	UA	48m	+	Hypotonia
Pt104	7m	UA	UA	UA	UA	UA	UA	41m	better	Hypotonia
Pt105	4m	UA	UA	UA	UA	UA	UA	28m	+	Hypotonia
Pt106	3m	UA	UA	UA	UA	UA	UA	105m	+	Hyperreflexia
Pt107	5m	UA	UA	UA	UA	UA	UA	15m	+	Hypotonia
Pt108	6m	UA	UA	UA	UA	UA	UA	37m	better	Hypotonia
Pt109	3m	UA	UA	UA	UA	UA	UA	43m	better	Hypotonia
Pt110	41m	UA	UA	UA	UA	UA	UA	2m	better	Hypotonia
Pt114	12m	UA	UA	UA	UA	UA	UA	40m	better	Hypotonia
Pt115	3m	UA	UA	UA	60m	-(4y)	Hypertonia, Joint Contracture	104m	+	Hypotonia
Pt116	18m	UA	UA	UA	UA	UA	UA	75m	better	Hyperreflexia
Pt117	2m	UA	UA	UA	UA	UA	UA	17m	better	Hyperreflexia
Pt118	69m	22m	+	Hypotonia	NA	NA	NA	NA	NA	NA

Case	First visit age	First Follow-up (2012.3)			Second Follow-up (2017.3)			Third Follow-up (2020.11)		
		Follow-up Interval	Nystagmus	Neurological findings	Follow-up Interval	Nystagmus	Neurological findings	Follow-up Interval	Nystagmus	Neurological findings
Pt119	4m	UA	UA	UA	71m	better	Hypotonia, Joint Contracture	115m	+	Hypotonia
Pt120	11m	1m	better	Hypotonia	NA	NA	NA	NA	NA	NA
Pt121	24m	46m	(-)5y	Joint Contracture	106m	-	Joint Contracture	NA	NA	NA
Pt122	24m	UA	UA	UA	UA	UA	UA	127m	+	Hyperreflexia
Pt124	3m	UA	UA	UA	UA	UA	UA	23m	better	Hyperreflexia
Pt125	6m	UA	UA	UA	35m		Joint Contracture, Ataxia	79m	better	Hyperreflexia
Pt126	6m	UA	UA	UA	56m		Joint Contracture, Ataxia	NA	NA	NA
Pt127	23m	67m	-(4y)	Hyperreflexia, Joint Contracture	NA	NA	NA	NA	NA	NA
Pt130	12m	UA	UA	UA	NA	NA	NA	76m	+	Hyperreflexia
Pt131	3m	8m	better	Hypotonia	NA	NA	NA	NA	NA	NA
Pt132	8m	10m	+	Hypotonia	NA	NA	NA	NA	NA	NA
Pt134	91m	40m	+	Hypotonia	NA	NA	NA	NA	NA	NA
Pt135	5m	UA	UA	UA	UA	UA	UA	2m	+	Hypotonia
Pt137	10m	60m	+	Hypotonia	NA	NA	NA	155m	better	Hyperreflexia
Pt138	6m	UA	UA	UA	UA	UA	UA	47m	better	Hypotonia
Pt139	13m	UA	UA	UA	56m	better	Joint Contracture	NA	NA	NA
Pt140	5m	UA	UA	UA	NA	NA	NA	10m	+	Hypotonia
Pt141	10m	8m	+	Joint Contracture	59m	better	Joint Contracture	NA	NA	NA

Legend: UA=Unavailable; NA= NA=not available due to lose to follow-up

Table S3. Clinical information of the 141 patients in our cohort

Case	Gender	Disease onset		Milestone						Other neurological features	PCS
		Nystagmus	Hypotonia	Head	sit	stand	walk	Recognize stranger	speak		
Pt1	M	5m		NA	NA	NA	NA	NA	NA	S	-
Pt2	M	10d	8m	24m	60m	NA	NA	NA	17m		2
Pt3	M	7d		4m	84m	90m	NA	36m	36m		1
Pt4	F	2m	12m	12m	NA	NA	NA	NA	NA		-
Pt5	M	2m	6m	6m	NA	NA	NA	36m	48m		3
Pt6	M	1m		9m	18m	48m	NA	6m	24m	HR	3
Pt7	M	6m	7m	7m	24m	36m	NA	7m	36m		-
Pt8	M	5d	7m	8m	24m	42m	NA	17m	24m	JC, ST	3
Pt9	M	35d	6m	6m	NA	NA	NA	8m	NA		-
Pt10	M	3m	5m	5m	9m	50m	50m	12m	11m	JC	4
Pt11	M	35d	1y	12m	NA	NA	NA	18m	18m	Se, S, JC, SD	2
Pt12	M	birth	26m	60m	NA	NA	NA	6m	72m		2
Pt13	M	7d	11m	36m	40m	48m	NA	12m	12m	JC	0
Pt14	M	birth	8m	8m	24m	48m	60m	60m	60m	JC	2
Pt15	M	4m	6m	6m	NA	NA	NA	30m	NA	JC	2
Pt16	M	15d	15m	4m	8m	36m	NA	36m	24m		4
Pt17	M	1m	18m	30m	36m		NA	5m	48m		2
Pt18	M	4m	8m	NA	NA	NA	NA	5m	NA		1
Pt19	M	3m	12m	12m	96m	84m	NA	12m	36m	SD	1
Pt20	M	8m	7m	8m	12m	36m	NA	36m	24m		5
Pt21	M	6m	18m	18m	24m	NA	NA	7m	24m		3
Pt22	M	15d		12m	NA	NA	NA	8m	NA	RE	2
Pt23	M	2m		NA	NA	NA	NA	5m	NA		2
Pt24	M	birth	7m	7m	86m	24m	24m	6m	24m	JC	2
Pt25	M	10d	9m	9m	NA	NA	NA	4m	12m	JC	2
Pt26	M	2m		4m	12m	NA	NA	8m	12m	S, JC	4
Pt27	M	3m	7m	24m	45m			6m	24m	S, SD	2
Pt28	M	2m	9m	3m	84m	NA	NA	12m	30m		4
Pt29	M	12m	12m	3m	12m	48m	NA	12m	86m	JC	3
Pt30	M	3m	6m	3m	7m	84m	96m	12m	12m	HR	4
Pt31	M	20d	7m	8m	12m	84m	120m	6m	24m	JC	4
Pt32	M	3m	5m	5m	NA	NA	NA	NA	NA		3
Pt33	M	3m	12m	5m	10m	24m	48m	5m	15m	JC	4
Pt34	M	1m	2m	8m	12m	12m	NA	10m	NA		4
Pt35	M	1m	14m	3m	12m	NA	NA	12m	24m	S, JC	4
Pt36	M	21d	6m	4m	9m	90m	NA	6m	12m	S, ST	4
Pt37	M	3m	6m	6m	6m	16m	NA	4m	12m		5
Pt38	M	15d	4m	10m	NA	NA	NA	NA	NA		3

Case	Gender	Disease onset		Milestone						Other neurological features	PCS
		Nystagmus	Hypotonia	Head	sit	stand	walk	Recognize stranger	speak		
Pt39	M	80d	7m	8m	NA	NA	NA	NA	10m		4
Pt40	M	4m	8m	14m	NA	NA	NA	12m	6m		3
Pt41	M	3d	7m	8m	NA	NA	NA	7m	11m		-
Pt42	M	-	15m	18m	21m	NA	NA	14m	12m	JC	4
Pt43	M	24d	9m	20m	NA	NA	NA	6m	24m	HR	2
Pt44	M	15d	12m	12m	NA	NA	NA	12m	24m	JC	2
Pt45	M	birth	18m	NA	NA	NA	NA	5m	NA	S	0
Pt46	M	2m	5m	4m	12m	20m	NA	NA	20m		-
Pt47	M	3m	5m	5m	36m	NA	NA	6m	24m	S, JC	3
Pt48	M	3m	9m	NA	NA	NA	NA	5m	NA		-
Pt49	M	3m	5m	NA	NA	NA	NA	NA	NA		-
Pt50	M	20d	6m	NA	NA	NA	NA	4m	NA		-
Pt51	M	7d	21m	NA	NA	NA	NA	NA	NA		-
Pt52	M	40d	10m	24m	NA	NA	NA	10m	24m	ST, RD	2
Pt53	M	45d	7m	14m	NA	NA	NA	6m	48m	S, JC	1
Pt54	M	7d	8m	NA	NA	NA	NA	18m	NA		-
Pt55	M	15d	10m	NA	NA	NA	NA	6m	NA		2
Pt56	M	1m	6m	4m	NA	NA	NA	4m	NA	ST	2
Pt57	M	1m	7m	8m	NA	NA	NA	NA	NA		-
Pt58	M	16m	9m	3m	9m	49m	NA	7m	12m	S	4
Pt59	M	23d	12m	12m	NA	NA	NA	8m	12m	S, SD	2
Pt60	M	birth	7m	8m	18m	NA	NA	7m	24m		-
Pt61	M	2m	6m	4m	12m	NA	NA	6m	8m		5
Pt62	M	24m	5m	5m	72m	NA	NA	12m	72m	DSK, ST	2
Pt63	M	8m	8m	4m	NA	NA	NA	NA	NA	S	2
Pt64	M	1m	6m	4m	NA	NA	NA	12m	NA	S, Se	2
Pt65	F	birth	7m	7m	12m	NA	NA	NA	NA		-
Pt66	M	birth	8m	9m	NA	NA	NA	5m	8m	S	2
Pt67	M	2m	9m	NA	NA	NA	NA	NA	NA		-
Pt68	M	birth	7m	NA	NA	NA	NA	NA	NA		-
Pt69	M	40d	9m	10m	NA	NA	NA	6m	NA		-
Pt70	M	5m	5m	12m	NA	NA	NA	7m	12m		-
Pt71	M	6m	5m	4m	48m	NA	NA	48m	48m	HR	2
Pt72	M	1m	7m	18m	NA	NA	NA	6m	6m		3
Pt73	M	15d	7m	6m	NA	NA	NA	12m	60m	S	2
Pt74	M	20d	9m	36m	NA	NA	NA	12m	8m	SD	2
Pt75	M	2m		NA	NA	NA	NA	NA	NA		-
Pt76	M	7d		NA	NA	NA	NA	36m	36m	S, RD	0

Case	Gender	Disease onset		Milestone						Other neurological features	PCS
		Nystagmus	Hypotonia	Head	sit	stand	walk	Recognize stranger	speak		
Pt77	M	2m	7m	8m	NA	NA	NA	9m	10m		4
Pt78	M	10d	5m	4m	NA	NA	NA	3m	NA	SD	1
Pt79	F	3d	6m	13m	NA	NA	NA	6m	22m	HR	1
Pt80	M	2m	12m	NA	NA	NA	NA	6m	NA		-
Pt81	M	1m	11m	NA	NA	NA	NA	5m	NA	SD, S	1
Pt82	M	1m	6m	12m	NA	NA	NA	10m	12m		3
Pt83	M	2m		NA	NA	NA	NA	NA	12m		3
Pt84	M	birth	12m	12m	NA	NA	NA	8m	12m		2
Pt85	M	birth		NA	NA	NA	NA	NA	NA		-
Pt86	M	3d	8m	20m	NA	NA	NA	10m	24m	SD	1
Pt87	M	4m		NA	NA	NA	NA	8m	NA	Se	3
Pt88	M	2m	7m	5m	30m	NA	NA	6m	12m		5
Pt89	M	3m	14m	6m	14m	NA	NA	NA	14m		5
Pt90	M	10d	6m	10m	NA	NA	NA	6m	12m	Se, S	2
Pt91	M	19d	12m	18m	30m	NA	NA	36m	36m	SD	3
Pt92	M	5m	6m	NA	NA	NA	NA	20m	NA	SD, S, RD	1
Pt93	M	6m	5m	NA	NA	NA	NA	6m	10m	S	3
Pt94	M	1m	5m	11m	NA	NA	NA	12m	25m		3
Pt95	M	6m	18m	4m	10m	NA	NA	6m	8m	S	4
Pt96	M	3d	2m	NA	NA	NA	NA	NA	NA	S	2
Pt97	F	2m	7m	NA	NA	NA	NA	12m	9m		3
Pt98	M	15d	3m	NA	NA	NA	NA	10m	NA	S, SD	1
Pt99	M	5m	5m	3m	15m	NA	NA	13m	18m		5
Pt100	M	5m	5m	3m	15m	NA	NA	12m	24m	HR	4
Pt101	M	2m	12m	12m	NA	NA	NA	6m	6m	S, HR	3
Pt102	M	15m	5m	5m	NA	NA	NA	12m	NA		3
Pt103	M	birth		NA	NA	NA	NA	NA	NA		-
Pt104	M	10m	12m	12m	NA	NA	NA	6m	11m	S	3
Pt105	M	2m	3m	8m	24m	NA	NA	6m	NA		5
Pt106	M	3m	4m	8m	NA	NA	NA	6m	20m	SD, S, HR	3
Pt107	M	1m	6m	NA	NA	NA	NA	6m	NA	SD, S	3
Pt108	F	birth	4m	NA	NA	NA	NA	5m	NA		1
Pt109	M	3m	3m	10m	12m	NA	NA	12m	24m		5
Pt110	M	7m	12m	6m	18m	NA	NA	12m	36m		4
Pt111	M	birth	127m	13m	NA	NA	NA	12m	NA		-
Pt112	M	birth	99m	3m	NA	NA	NA	36m	40m		-
Pt113	M	birth	19m	NA	NA	NA	NA	NA	NA		-
Pt114	M	1m	12m	NA	NA	NA	NA	12m	NA		2
Pt115	M	20d	3m	12m	NA	NA	NA	5m	72m	SD, S	1

Case	Gender	Disease onset		Milestone						Other neurological features	PCS
		Nystagmus	Hypotonia	Head	sit	stand	walk	Recognize stranger	speak		
Pt116	F	birth	18m	5m	9m	26m	29m	6m	12m	HR	3
Pt117	M	1m		NA	NA	NA	NA	NA	NA	SD, S, HR	1
Pt118	M	birth		NA	NA	NA	NA	NA	NA	Se	0
Pt119	M	birth		NA	NA	NA	NA	7m	NA	SD, S, JC, RD	0
Pt120	M	birth		6m	NA	NA	NA	7m	NA		1
Pt121	M	15d	24m	4m	36m	9m	48m	8m	12m	JC, RE	3
Pt122	M	1m		7m	NA	NA	NA	8m	12m	HR	2
Pt123	M	3m	11m	5m	NA	NA	NA	NA	NA		-
Pt124	M	3m	3m	NA	NA	NA	NA	12m	12m	HR	2
Pt125	M	1m		NA	NA	NA	NA	30m	NA	S, SD, HR, Se, JC	1
Pt126	M	1m	6m	24m	NA	NA	NA	NA	60m	JC	1
Pt127	M	12m		NA	NA	NA	NA	48m	72m	HR, RE, JC	1
Pt128	M	birth	18m	4m	8m	NA	NA	6m	9m		-
Pt129	M	3d	12m	6m	NA	NA	NA	NA	NA		-
Pt130	M	1m	12m	36m	NA	NA	NA	8m	12m	HR	2
Pt131	M	40d	3m	18m	24m	NA	NA	6m	24m		4
Pt132	M	2m		14m	NA	NA	NA	4m	8m	S	2
Pt133	M	1m	6m	56m	NA	NA	NA	NA	NA		-
Pt134	M	2m		5m	24m	NA	NA	6m	18m		5
Pt135	M	2m	2m	NA	NA	NA	NA	NA	NA	SD, S	1
Pt136	M	4m	12m	NA	NA	NA	NA	6m	NA		-
Pt137	M	birth	10m	71m	NA	NA	NA	48m	48m	HR, S	0
Pt138	M	20d	6m	NA	NA	NA	NA	NA	NA	SD, S, Se	1
Pt139	M	1m	13m	NA	NA	NA	6m	NA	13m	JC, Se	1
Pt140	M	10m	5m	NA	NA	NA	NA	NA	NA	SD, S	1
Pt141	M	10m		NA	NA	NA	NA	6m	NA	JC	1

Legend: M=male, F=female, NA=not available due to lose to follow-up, S=stridor, HR=hyperreflexia, JC=joint contracture, ST=spasticity tetraparesis, Se=seizure, SD=swallowing disorder, RD=respiration difficulty.
